# Supplementary material for: Acceptability and usability of a mobile application for management and surveillance of vector-borne diseases in Colombia: An implementation study
Source: PLoS One. 2020 May 29;15(5):e0233269. doi: 10.1371/journal.pone.0233269 (PMC7259752; doi:10.1371/journal.pone.0233269)
Supplement: S1 File — (PDF) [file pone.0233269.s001.pdf]

S1 Evaluation form (English version)

EVALUATION FORM - APPLICATION

Modified from: Mobile Application rating Scale

1. Do you know the updated guidelines from the Colombian Ministry of Health regarding Dengue, Chikungunya, and Zika management?
    1. You don't know them
    2. You know they exist, but you haven't read them
    3. You have read them
    4. You have read and understood them
    5. You have understood and applied them during your clinical practice

2. According to your knowledge, do you consider that the information provided in the application follows the management guidelines?
    1. Does not apply
    2. Strongly disagree
    3. Disagree
    4. Somewhat agree
    5. Strongly agree

The rating scale evaluates the quality of the application in 5 dimensions. All items are rated on a 5-point scale, where 1 is inappropriate, and 5 is excellent. Please check, in the answer sheet, the circle that corresponds to the answer that most accurately represents the quality of the application component you are rating.

SECTION A: SCIENTIFIC AND CLINICAL INFORMATION

3. The application has specific objectives, measurable and achievable (specified in the application)?
    1. The application does not have any possibility to achieve the declared objectives.
    2. The description stated some objectives, but the application has very little chance of achieving them.
    3. OK. The application has clear objectives that can be achievable.
    4. The objectives of the application are specified, measurable, and achievable.
    5. The application has specific and measurable goals, which are highly likely to be achieved.

4. Quality of the information: Is the content of the application correct; is it well written and relevant for the application's objective?

    1. Irrelevant content / inappropriate / incoherent / incorrect
    2. Bad content; not relevant / appropriate / coherent / may be incorrect
    3. Moderately relevant / appropriate / coherent / seems correct
    4. Relevant / appropriate / coherent / correct

    5. Strongly relevant, adequate, coherent and correct

5. Quantity of information: Is the information within the app comprehensive but concise?
    1. The information is minimal or overwhelming
    2. The information is insufficient and possibly overwhelming
    3. Good, but is not comprehensive or concise
    4. It offers a wide range of information, has some gaps or unnecessary details; or does not have links to more information and resources
    5. The information is comprehensive and concise; contains links to more information and resources

6. Visual information: Is the visual explanation of concepts (through charts / graphs /images / videos / etc.) clear, logic and correct?
    1. It is not clear / it is confusing / incorrect / it is necessary but it is missing
    2. Mostly unclear / confusing / it is incorrect
    3. It is OK, but it is often unclear / confusing / incorrect
    4. Mostly clear / logical / correct; with insignificant problems
    5. Perfectly clear / logical / correct

7. Credibility: Does the information within the application come from a credible source (specified within the application)?
    1. It identifies the sources, but its legitimacy / reliability is questionable (commercial, with conflicts of interest)
    2. It seems to come from a legitimate source , but it cannot be verified (e.g., does not have a website)
    3. Developed by a small NGO / institution (hospital / center, etc.) / specialized business
    4. Developed by the government, a university or as the previous point but greater dimensions
    5. Developed with national funds from the national government for research (e.g., Australian Research Council)

8. References: Are the bibliographic references clear and exposed in the application?
    1. Strongly disagree
    2. Somewhat disagree
    3. I don't know
    4. Somewhat agree
    5. Strongly agree

9. Knowledge: Do you consider that this application can improve your knowledge / understanding of the approach and management of dengue, Zika, and chikungunya infections?
    1. Strongly disagree
    2. Somewhat disagree
    3. I don't know
    4. Somewhat agree
    5. Strongly agree

10. I had to learn many things before I could use the application
    1. Strongly disagree
    2. Somewhat disagree
    3. I don't know
    4. Somewhat agree
    5. Strongly agree

SECTION B: USER ENGAGEMENT

Evaluate where the application is fun, interesting, customizable, interactive (for example, it sends alerts, messages, reminders, feedback, enables sharing), and well-targeted to the audience.

11. Interest: Is the application interesting to use? Does it use any strategy to increase engagement by interestingly presenting the content?
    1. It is not interesting at all
    2. Not very interesting
    3. OK, neither interesting nor uninteresting
    4. Moderately interesting
    5. Very interesting, would use it again

12. Interactivity: Does the application allow the user input, provide feedback?
    1. There are no interactive features and / or no response to the user input
    2. Insufficient interactivity or feedback
    3. Basic interactive features and work properly
    4. Offers a variety of interactive features / comments / user input options
    5. Very high level of responsiveness / feedback / user input option

13. Target group: Is the content of the application (visual information, language, and design) appropriate for the end-user (healthcare workers)?
    1. It is completely inapropriate / unclear / confusing
    2. Mostly innapropriate /confusing
    3. Acceptable content, but not specifically designed for the target audience
    4. Properly targeted, with negligible content issue
    5. Perfectly targeted, no content issues

SECTION C: FUNCTIONALITY

Evaluate the operation of the application (navigation / flow logic / gestural application design)

14. Performance: How accurately / fast do the application's features and components (buttons / menus) work?
    1. The app is broken; no / insufficient / inappropriate (e.g., crashes / bugs, etcetera)
    2. Some features work correctly, but with delayed or contain major technical problems
    3. The app works well in general, with some technical issues (e.g., slow at times)
    4. Mostly functional with minor / insignificant problems

5. Perfect and timely function of the app; there are no technical errors.

15. Ease of use: Is it easy to learn how to use the application; (menus/labels/clear icons and instructions)?
    1. There are no instructions, or they are limited; menu labels/icons are confused or complicated
    2. Easy to use after a long time / effort
    3. Easy to use after some time / effort
    4. Easy to use / clear instructions
    5. Easy to use immediately (intuitive and simple)

16. Navigation: Does moving between screens make sense views (screens) is logic/precise/appropriate/uninterrupted; Do all links work?

    1. The different sections within the application are not logically connected or are confusing, and navigation is difficult
    2. Navigation is easy after a long time / effort
    3. You can navigate after some time / effort
    4. Easy navigation with insignificant errors between views (screens)
    5. Navigation between views (screens) perfectly logical, easy, clear and intuitive throughout the interaction, offers shortcuts

17. Gestural design: Are the interactions (Taps/Swipes/Pinches/Scroll) coherent and intuitive in all views (screens)?
    1. Completely inconsistent / confusing
    2. Often inconsistent / confusing
    3. OK. With some inconsistencies / confusing elements
    4. Very consistent / intuitive with insignificant problems
    5. Perfectly consistent and intuitive

SECTION D: AESTHETICS - GRAPHIC DESIGN

Evaluate the visual appeal of the application, color scheme, and consistency in style.

18. Design: Is the layout and size of buttons / icons / menus / screen content adequate, and does it allow you to use the zoom if necessary?
    1. Very bad design (messy, some options are impossible to select / locate / see / read, does not optimize device screen)
    2. Bad design (random, not clear, options are difficult to select / locate / see / read)
    3. Satisfactory. Few issues with option selection / positioning / seeing / reading, minor issues with screen size
    4. Mostly clear, the options are easy to select / locate / see / read)
    5. Professional, simple, clear, logically organized, the device's screen is optimized. Each component has a purpose

19. Graphics: How high are the quality / resolution of the graphics used for buttons / icons / menus / content?
    1. Inferior visual design (disproportionate, inconsistent style)

2. Low quality and graphic resolution. Low quality visual design (disproportionate, inconsistent style)
3. Moderate quality of the graphics and visual design of (generally consistent in style)
4. High quality / resolution of graphics and visual design - mostly proportionate and consistent instyle
5. Very high quality / resolution of graphics and visual design- proportionate, consistent in style throughout the entire application

20. Visual appeal: How does the application look?
    1. No visual appeal, unpleasant to look at, poorly designed - mismatched colors
    2. Little visual appeal - poorly designed, color misuse, visually boring
    3. Some visual appeal - average, neither pleasant, nor unpleasant
    4. High level of visual appeal - seamless graphics - consistent and professional design
    5. Like the above + very attractive, easy to remember, stands out; use of color improves the application / the menus

SETCION E: IMPACT

Evaluates the impact on patient management from the health professional perspective

21. Intention to change: This application can increase motivation / intention to improve adherence to dengue, Zika and chikungunya management guidelines
    1. Strongly disagree
    2. Somewhat disagree
    3. I don't know
    4. Somewhat agree
    5. Strongly agree

22. Attitudes: Attitudes towards approach and management of patients with dengue, Zika and, chikungunya are likely to change
    1. Strongly disagree
    2. Somewhat disagree
    3. I don't know
    4. Somewhat agree
    5. Strongly agree

23. This application has the potential to be efficiently introduced in multiple emergency services at different levels of healthcare attention in Colombia
    1. Strongly disagree
    2. Somewhat disagree
    3. I don't know
    4. Somewhat agree
    5. Strongly agree

SECTION F: SUBJECTIVE QUALITY EVALUATION

Evaluates the acceptability of the application by the health professional

24. Would you recommend this application to people who might benefit from it?
    1. No, not at all. I would not recommend this application to anyone
    2. There are very few people to whom I would recommend this application
    3. There are several people I would recommend this app to
    4. There are many people I would recommend this app to
    5. I would recommend this application to everyone

25. I think I would use this application frequently
    1. Strongly disagree
    2. Somewhat disagree
    3. I don't know
    4. Somewhat agree
    5. Strongly agree

26. Would you pay for this application?
    1. No
    2. .
    3. Maybe
    4. .
    5. Yes

27. What is your overall (star) rating of the application?
    1. ★ One of the worse applications I have used
    2. ★ ★
    3. ★ ★ ★ Average / mediocre
    4. ★ ★ ★ ★
    5. ★ ★ ★ ★ ★ One of the best applications I have used
