# Supplementary material for: Acceptability and usability of a mobile application for management and surveillance of vector-borne diseases in Colombia: An implementation study
Source: PLoS One. 2020 May 29;15(5):e0233269. doi: 10.1371/journal.pone.0233269 (PMC7259752; doi:10.1371/journal.pone.0233269)
Supplement: S2 File — (PDF) [file pone.0233269.s002.pdf]

S3. Evaluation form (Spanish version)

FORMATO DE EVALUACIÓN – APLICACIÓN

Modificado de: Mobile Application rating Scale

1. ¿Conoce usted las últimas guías del Ministerio de Salud para el manejo de Dengue, Chikungunya y Zika?
    1. No las conoce
    2. Sabe que existen pero no las ha leído
    3. Las ha visto
    4. Las ha leído y comprendido
    5. Las ha comprendido y aplicado en su práctica clínica

2. De acuerdo con su conocimiento ¿considera usted que la información brindada por la aplicación está acorde con las guías de manejo?
    1. No Aplica
    2. Completamente en desacuerdo
    3. En desacuerdo
    4. Algo de acuerdo
    5. Completamente de acuerdo

La escala de calificación evalúa la calidad de la aplicación en 5 dimensiones. Todos los ítems son calificados en una escala de 5 puntos, donde 1 es inadecuado y 5 es excelente. Por favor marque, en la hoja de respuestas, el círculo que corresponde a la respuesta que representa con mayor precisión la calidad del componente de la aplicación que está calificando.

SECCIÓN A: INFORMACIÓN / BASES CIENTÍFICAS Y CLÍNICAS

3. ¿La aplicación tienen objetivos específicos, medibles y alcanzables (especificados en la aplicación)?
    1. La aplicación no tiene ninguna posibilidad de lograr sus objetivos declarados
    2. La descripción enumera algunos objetivos, pero la aplicación tiene muy pocas posibilidades de alcanzarlos
    3. OK. Aplicación tiene objetivos claros, que pueden ser alcanzables.
    4. Los objetivos de la aplicación son especificados claramente, medibles y alcanzables
    5. Aplicación tiene metas específicas y medibles, que son altamente probables de alcanzar.

4. Calidad de la información: ¿El contenido de la aplicación es correcto, está bien escrito y es relevante al tema/objetivo de la misma?
    1. Contenido irrelevante / inapropiado / incoherente / incorrecto
    2. Contenido malo; poco relevante / apropiado / coherente/ puede ser incorrecto
    3. Moderadamente relevante / apropiado / coherente / parece correcto

4. Relevante / apropiado / coherente / correcto
5. Muy relevante, adecuada, coherente y correcto

5. Cantidad de información: ¿Cómo considera la cobertura de del tema dentro del alcance de la aplicación; la información es completa pero concisa?
    1. La información es mínima o abrumadora
    2. La información es insuficiente o posiblemente abrumadora
    3. Bien, pero no es completa o concisa
    4. Ofrece una amplia gama de información, tiene algunas lagunas o detalles innecesarios; o no tiene enlaces a más información y recursos.
    5. La información es exhaustiva y concisa; contiene enlaces a más fuentes y recursos

6. Información visual: ¿Es la explicación visual de conceptos (a través de cuadros / gráficos / imágenes / vídeos, etc.) clara, lógica y correcta?
    1. No es clara / es confusa / incorrecta/ es necesaria pero falta
    2. En su mayoría no es clara / confusa / es incorrecta
    3. Está bien, pero a menudo es poco clara / confusa / incorrecta
    4. En su mayoría clara/ lógica / correcta; con problemas insignificantes
    5. perfectamente clara / lógica / correcta

7. Credibilidad: ¿La aplicación proviene de una fuente legítima (especificada al interior de la aplicación)?
    1. Identifica las fuentes, pero la legitimidad/confiabilidad de estas es cuestionable (comercial, con conflictos de interés)
    2. Parece provenir de una fuente legítima, pero esta no puede ser verificada (Ej. no tiene página web)
    3. Desarrollado por pequeña ONG / institución (hospital / centro, etc.) / negocio especializado
    4. Desarrollado por el gobierno, una universidad o como el punto anterior pero de mayores dimensiones
    5. Desarrollado con fondos nacionales del gobierno nacional para investigación (Ej. Consejo de investigación de Australia)

8. Referencias: ¿Las referencias bibliográficas son claras y se encuentran expuestas en la aplicación?
    1. Completamente en desacuerdo
    2. Algo en desacuerdo
    3. No sé
    4. Algo de acuerdo
    5. Completamente de acuerdo

9. Conocimiento: ¿Considera que esta aplicación puede aumentar su conocimiento/compresión sobre el enfoque y manejo de infección por dengue, chikungunya y zika?
    1. Completamente en desacuerdo
    2. Algo en desacuerdo
    3. No sé
    4. Algo de acuerdo

5. Completamente de acuerdo

10. Tuve que aprender muchas cosas antes de poder utilizar la aplicación
    1. Completamente en desacuerdo
    2. Algo en desacuerdo
    3. No sé
    4. Algo de acuerdo
    5. Completamente de acuerdo

SECCIÓN B: CAPTACIÓN DEL USUARIO

Evalúa si la aplicación es divertida, interesante, personalizable, interactiva (por ejemplo, envía alertas, mensajes, recordatorios, la retroalimentación, permite el uso compartido) y bien dirigido a la audiencia

11. Interés: ¿Es la aplicación interesante utilizar? ¿Utiliza alguna estrategia para aumentar la participación mediante la presentación de su contenido de una manera interesante?
    1. No es interesante en absoluto
    2. Muy interesante
    3. OK, Ni interesante ni carente de interés
    4. Moderadamente interesante
    5. Muy interesante, volvería a utilizar

12. Interactividad: ¿Permite al usuario la entrada de datos y proporciona retroalimentación?
    1. No hay características interactivas y / o no hay respuesta a la interacción del usuario
    2. Interactividad o retroalimentación insuficiente
    3. Características interactivas básicas y funcionan adecuadamente
    4. Ofrece una variedad de características / comentarios / opciones de ingreso de información para el usuario
    5. Muy alto nivel de respuesta / retroalimentación / ingreso de datos

13. Grupo objetivo: ¿Es el contenido de la aplicación (información visual, lenguaje, y el diseño) apropiado para el usuario final (Personal de salud)?
    1. Es totalmente inapropiada / incierto / confusa
    2. En su mayoría inapropiado / confuso
    3. Contenido Aceptable, pero no dirigida al usuario final
    4. Orientada adecuadamente, con problemas insignificantes de contenido
    5. perfectamente dirigida, Sin problemas de contenido

SECCIÓN C: FUNCIONALIDAD

Evalúa el funcionamiento de la aplicación (navegación, lógica de flujo, diseño de la aplicación gestual)

14. Rendimiento: ¿Con qué precisión/rapidez trabajan las características (funciones) y componentes (Botones / menús) de la aplicación?
    1. La App está dañada; las respuestas son nulas/ insuficientes/inapropiadas (Ej. se cierra / tiene errores, etc.)

2. Algunas funciones trabajan adecuadamente, pero con retraso o contiene problemas técnicos mayores
3. La App funciona bien en general, con algunos problemas técnicos (Ej. lento en ocasiones)
4. Muy funcional con problemas menores / insignificantes
5. Función perfecta y oportuna ; no hay errores técnicos

15. Facilidad de uso: ¿Es fácil aprender a usar la aplicación; (los menús/etiquetas/iconos e instrucciones son claras?
    1. No hay Instrucciones o son limitadas; Las etiquetas /iconos de menú son confusas o Complicadas
    2. Fácil de usar después de mucho tiempo/esfuerzo
    3. Fácil de usar después de algún tiempo/esfuerzo
    4. Fácil de usar/Instrucciones claras
    5. Fácil de usar inmediatamente (Intuitiva y sencilla)

16. Navegación: ¿Los cambios entre las vistas (Pantallas) son lógicos/precisos /apropiados/ininterrumpidos; Funcionan todos los enlaces?
    1. Las diferentes secciones dentro de la aplicación no se conectan lógicamente o son confusos y la navegación es difícil
    2. La navegación es fácil después de mucho tiempo / esfuerzo
    3. Se puede navegar después de algún tiempo/esfuerzo
    4. Fácil navegación con errores insignificantes entre las vistas (pantallas)
    5. Navegación entre vistas (pantallas) perfectamente lógica, fácil, clara e intuitiva a lo largo de la interacción, ofrece accesos directos

17. Diseño gestual: ¿Las interacciones (Taps/Swipes/Pinches/Scroll) son coherentes e intuitivos en todas las vistas/pantallas?
    1. Completamente inconsistente / confuso
    2. A menudo inconsistente / confuso
    3. OK. con algunas inconsistencias / elementos confusos
    4. Muy coherente / intuitiva con problemas insignificantes
    5. Perfectamente coherente e intuitiva

SECCIÓN D: ESTÉTICA – DISEÑO GRÁFICO

Evalúa el atractivo visual de la aplicación, esquema de color y consistencia en el estilo.

18. Diseño: La disposición y tamaño de los botones / iconos / menús / contenido en la pantalla es adecuada y permite la utilización de zoom si es necesario?
    1. Diseño muy malo (desordenado, algunas opciones son imposibles de seleccionar / localizar / ver / lee, no optimiza la pantalla del dispositivo)
    2. Diseño malo (al azar, no es claro, opciones difíciles de seleccionar / localizar / ver / leer)
    3. Satisfactorio. Pocos problemas con la selección / posicionamiento / visión / lectura de opciones, problemas menores con el tamaño de pantalla.
    4. Mayormente despejado, las opciones son fáciles de seleccionar / localizar / ver / leer.

5. Profesional, simple, claro, ordenado, lógicamente organizado, la pantalla del dispositivo se optimiza. Cada componente tiene un propósito.

19. Gráficos: ¿Qué tan alto es la calidad / resolución de los gráficos utilizados para los botones / iconos / menús / contenido?
    1. Muy pobre diseño visual (desproporcionada, estilo completamente inconsistente)
    2. Pobre calidad y resolución gráfica. Diseño visual de pobre calidad (desproporcionado, estilo inconsistente.
    3. Gráficos y diseño visual de calidad moderada (generalmente consistente en su estilo)
    4. Alta calidad/resolución d graficos diseño visual - en su mayoría proporcionado y de estilo consistente.
    5. Muy alta calidad / resolución de gráficos y diseño visual - proporcional, estilo consistente en toda la aplicación.

20. Atractivo visual: ¿Qué tan bien se ve la aplicación?
    1. Sin atractivo visual, desagradable a la vista, mal diseñada - los colores chocan - no coinciden
    2. Poco atractivo visual - mal diseñada, mal uso del color, visualmente aburrida
    3. Algún atractivo visual - Promedio, ni agradable ni desagradable
    4. Alto nivel de atractivo visual - gráficos sin costura - diseño consistente y profesional
    5. Como el anterior + muy atractivo, fácil de recordar, se destaca; uso del color mejora la aplicación/ los menús

SECCIÓN E: IMPACTO

Evalúa el impacto en el manejo del paciente desde la perspectiva del profesional de la salud

21. Intención de cambio: Esta aplicación puede aumentar la motivación/intención de mejorar adherencia a guías de manejo de dengue, zika y chikungunya
    1. Completamente en desacuerdo
    2. Algo en desacuerdo
    3. No sé
    4. Algo de acuerdo
    5. Completamente de acuerdo

22. Actitudes: Es probable que cambien las actitudes hacia el enfoque y manejo de pacientes con dengue, zika y chikungunya
    1. Completamente en desacuerdo
    2. Algo en desacuerdo
    3. No sé
    4. Algo de acuerdo
    5. Completamente de acuerdo

23. Esta aplicación tiene potencial para ser introducida de manera eficiente en múltiples servicios de urgencias de diferentes niveles de atención en Colombia
    1. Completamente en desacuerdo
    2. Algo en desacuerdo
    3. No sé
    4. Algo de acuerdo

5. Completamente de acuerdo

SECCIÓN F: EVALUACIÓN SUBJETIVA DE CALIDAD

Evalúa la aceptabilidad de la aplicación por parte del usuario

24. ¿Recomendaría esta aplicación a personas que podrían beneficiarse de ella?
    1. No, en absoluto. No recomendaría esta aplicación a nadie
    2. Hay muy pocas personas a quienes recomendaría esta aplicación
    3. Hay varias personas a quienes recomendaría la aplicación
    4. Hay muchas personas a quienes recomendaría esta aplicación
    5. Sin duda, recomendaría esta aplicación a todo el mundo

25. Creo que utilizaría esta aplicación frecuentemente
    1. Completamente en desacuerdo
    2. Algo en desacuerdo
    3. No sé
    4. Algo de acuerdo
    5. Completamente de acuerdo

26. ¿Usted pagaría por esta aplicación?
    1. No
    2. .
    3. Tal vez
    4. .
    5. Si

27. ¿Cuál es su valoración global (por estrellas) de la aplicación?
    1. ★ Una de las peores aplicaciones que he usado
    2. ★ ★
    3. ★ ★ ★Promedio/mediocre
    4. ★ ★ ★ ★
    5. ★ ★ ★ ★ ★Una de las mejores aplicaciones que he usado

FORMATO PARA EVALUACIÓN DE APLICACIÓN MÓVIL

HOJA DE RESPUESTAS

CÓDIGO EVALUADOR: __________________________ CARGO: ______________________________
FECHA: _______________________________________ EDAD: _______
SISTEMA OPERATIVO: __________________________

Sección	Pregunta	1	2	3	4	5
NA	1					
	2					
A	3					
	4					
	5					
	6					
	7					
	8					
	9					
	10					
B	11					
	12					
	13					
C	14					
	15					
	16					
	17					
D	18					
	19					
	20					
E	21					
	22					
	23					
F	24					
	25					
	26					
	27					

Total categoría de Información (A):	
Total categoría de captación del usuario (B):	
Total categoría de funcionalidad (C):	
Total categoría de estética y diseño gráfico (D):	
Total categoría de impacto (E):	
Total evaluación subjetiva de calidad (F):	
Total evaluación:	
