# Supplementary material for: Acceptability and usability of a mobile application for management and surveillance of vector-borne diseases in Colombia: An implementation study
Source: PLoS One. 2020 May 29;15(5):e0233269. doi: 10.1371/journal.pone.0233269 (PMC7259752; doi:10.1371/journal.pone.0233269)
Supplement: S1 Dataset — (PDF) [file pone.0233269.s003.pdf]

S2. General practitioner individual score

GP	Q1	Q2	Q3	Q4	Q5	Q6	Q7	Q8	Q9	Q10	Q11	Q12	Q13	Q14	Q15	Q16	Q17	Q18	Q19	Q20	Q21	Q22	Q23	Q24	Q25
1	1	4	3	4	4	3	4	3	5	1	5	4	4	5	5	5	5	5	4	4	5	5	5	5	5
2	2	4	4	3	4	4	4	2	5	1	4	5	4	4	5	5	5	5	4	4	4	5	3	4	5
3	2	1	5	4	4	4	4	3	5	2	5	5	4	4	4	5	5	4	4	4	3	5	5	4	4
4	2	4	4	5	4	5	4	5	5	1	5	4	5	5	5	5	5	5	5	4	5	5	5	5	5
5	2	5	3	4	4	3	2	5	5	1	4	3	4	4	5	4	4	4	3	4	5	5	5	4	4
6	3	5	4	5	5	4	5	3	5	1	5	4	4	5	4	5	5	4	5	4	5	5	5	5	5
7	3	4	2	4	5	3	4	4	4	2	3	3	3	4	5	4	3	3	4	3	2	2	4	4	2
8	3	5	4	5	5	5	4	4	5	1	4	4	5	4	5	4	5	5	5	5	5	5	5	5	4
9	3	1	3	4	4	4	3	5	5	2	5	3	5	5	3	4	4	5	5	5	5	5	5	5	4
10	3	1	5	5	5	5	4	4	5	4	5	5	4	5	4	5	5	5	5	5	4	4	3	5	4
11	3	1	3	4	4	4	3	4	5	2	4	3	4	5	4	4	5	4	4	4	5	4	4	4	5
12	4	5	4	4	5	4	4	-	3	5	4	5	5	4	4	4	4	5	4	5	5	5	5	5	5
13	4	5	1	4	4	4	3	4	5	1	5	4	4	4	5	5	5	4	5	5	5	4	5	4	4
14	4	4	3	4	4	4	3	3	4	2	5	4	4	4	4	4	3	3	3	3	5	4	5	4	4
15	5	5	5	5	5	4	4	5	4	5	1	5	5	4	5	5	4	5	5	5	4	4	5	3	4
16	5	5	4	5	5	4	4	5	5	1	5	4	5	4	4	5	5	5	4	5	5	5	5	5	5
17	5	5	5	4	4	4	3	5	5	2	5	4	4	4	5	5	5	4	3	4	5	2	5	5	4
18	5	4	4	4	4	4	4	4	4	4	4	4	4	4	5	4	4	4	4	4	4	4	4	4	4
19	5	5	4	4	4	5	4	5	5	1	5	5	4	4	5	5	5	4	4	4	5	5	5	5	5
20	5	5	4	5	5	5	4	5	5	2	4	4	4	5	5	5	5	5	4	5	3	4	5	5	3

GP: General Practitioner
