# Supplementary material for: Acceptability and usability of a mobile application for management and surveillance of vector-borne diseases in Colombia: An implementation study
Source: PLoS One. 2020 May 29;15(5):e0233269. doi: 10.1371/journal.pone.0233269 (PMC7259752; doi:10.1371/journal.pone.0233269)
Supplement: Attachment — Submitted filename: Response to Reviewers.docx [file pone.0233269.s004.docx]

Editors

*PLOS ONE*

February 17, 2020

Dear Dr. Samy,

We thank you and the reviewers for a thorough reading and constructive review of our manuscript and for the opportunity to revise and resubmit. We are pleased to submit the improved research article, including a new version of the mobile app interface shown in the figures for your consideration in the PLOS ONE. On the following pages, you will find our response to the editor and reviewer's comments. On behalf of my co-authors, I thank you for your consideration of this resubmission. We appreciate your time and look forward to your response.

Sincerely,

Sarita Rodríguez R, M.D. (corresponding author) [sarita.rodriguez@fvl.org.co](mailto:sarita.rodriguez@fvl.org.co), +573173820407

*PONE-D-19-31941
Acceptability and usability of a mobile application for management and surveillance of vector-borne diseases in Colombia: An implementation study
PLOS ONE

Dear Dr Rodriguez,

Thank you very much for submitting your manuscript "Acceptability and usability of a mobile application for management and surveillance of vector-borne diseases in Colombia: An implementation study" (#PONE-D-19-31941) for review by PLOS ONE. As with all papers submitted to the journal, your manuscript was fully evaluated by academic editor (myself) and by independent peer reviewers. The reviewers appreciated the attention to an important health topic, but they raised substantial concerns about the paper that must be addressed before this manuscript can be accurately assessed for meeting the PLOS ONE criteria. Therefore, if you feel these issues can be adequately addressed, we invite you to submit a revised version of the manuscript that addresses the points raised during the review process. We can’t, of course, promise publication at that time.

We would appreciate receiving your revised manuscript by Feb 17 2020 11:59PM. When you are ready to submit your revision, log on to https://www.editorialmanager.com/pone/ and select the 'Submissions Needing Revision' folder to locate your manuscript file.

If you would like to make changes to your financial disclosure, please include your updated statement in your cover letter.

To enhance the reproducibility of your results, we recommend that if applicable you deposit your laboratory protocols in protocols.io, where a protocol can be assigned its own identifier (DOI) such that it can be cited independently in the future. For instructions see:* [*http://journals.plos.org/plosone/s/submission-guidelines#loc-laboratory-protocols*](http://journals.plos.org/plosone/s/submission-guidelines#loc-laboratory-protocols)

*Please include the following items when submitting your revised manuscript:*

- *A rebuttal letter that responds to each point raised by the academic editor and reviewer(s). This letter should be uploaded as separate file and labeled 'Response to Reviewers'.*
- *A marked-up copy of your manuscript that highlights changes made to the original version. This file should be uploaded as separate file and labeled 'Revised Manuscript with Track Changes'.*
- *An unmarked version of your revised paper without tracked changes. This file should be uploaded as separate file and labeled 'Manuscript'.*

*Please note while forming your response, if your article is accepted, you may have the opportunity to make the peer review history publicly available. The record will include editor decision letters (with reviews) and your responses to reviewer comments. If eligible, we will contact you to opt in or out.*

*We look forward to receiving your revised manuscript.

Kind regards,

Abdallah M. Samy, PhD
Academic Editor
PLOS ONE*

*Journal Requirements:*

*When submitting your revision, we need you to address these additional requirements:*

*1. Please ensure that your manuscript meets PLOS ONE's style requirements, including those for file naming. The PLOS ONE style templates can be found at http://www.plosone.org/attachments/PLOSOne_formatting_sample_main_body.pdf and http://www.plosone.org/attachments/PLOSOne_formatting_sample_title_authors_affiliations.pdf*

We have reformatted the manuscript according to the above style guidelines. These changes include the authors' affiliation formatting and contribution conventions.

***Reviewers' comments:***
*Reviewer's Responses to Questions

Comments to the Author

1. Is the manuscript technically sound, and do the data support the conclusions?

The manuscript must describe a technically sound piece of scientific research with data that supports the conclusions. Experiments must have been conducted rigorously, with appropriate controls, replication, and sample sizes. The conclusions must be drawn appropriately based on the data presented. 

Reviewer #1: Partly*

*2. Has the statistical analysis been performed appropriately and rigorously? 

Reviewer #1: No*

*3. Have the authors made all data underlying the findings in their manuscript fully available?

The* [*PLOS Data policy*](http://www.plosone.org/static/policies.action#sharing) *requires authors to make all data underlying the findings described in their manuscript fully available without restriction, with rare exception (please refer to the Data Availability Statement in the manuscript PDF file). The data should be provided as part of the manuscript or its supporting information, or deposited to a public repository. For example, in addition to summary statistics, the data points behind means, medians and variance measures should be available. If there are restrictions on publicly sharing data—e.g. participant privacy or use of data from a third party—those must be specified.

Reviewer #1: No*

We appreciate this observation; thus, to compliant the PLOS Data Policy, we will make raw data concerning the results of the FeverDx usability and acceptability evaluation that supports our work fully available as part of the supporting information.

*4. Is the manuscript presented in an intelligible fashion and written in standard English?

PLOS ONE does not copyedit accepted manuscripts, so the language in submitted articles must be clear, correct, and unambiguous. Any typographical or grammatical errors should be corrected at revision, so please note any specific errors here.

Reviewer #1: Yes*

5*. Review Comments to the Author*

*Please use the space provided to explain your answers to the questions above. You may also include additional comments for the author, including concerns about dual publication, research ethics, or publication ethics. (Please upload your review as an attachment if it exceeds 20,000 characters)*

*Reviewer #1: The authors present the results of a pilot acceptability and usability mobile application to help GPs with diagnosing arbovirus and leptospirosis (although it is ignored in most of the text which is centered on arboviruses), and allow them to report cases directly for surveillance purposes. The paper is well written, and I found the mhealth application interesting, useful and well-though. However, the study presented here is very limited, biased and, although it is a good first step, the study needs to be expanded in order to warrant a manuscript rather than a short communication. I would suggest expanding the study to other settings and the sample size.*

We thank you for this comment. We agree that in some sentences in the abstract and the introduction, it seemed that we focus on arboviruses and leptospirosis. However, we included leptospirosis and other infections in our environment due to the frequency of presentation and that these infections are part of the differential diagnosis of dengue, Zika, and chikungunya. Consequently, we replaced "Leptospirosis" from Lines 52, 185, 457 for "other common infections" in the file “Manuscript with track changes”.

We share your concerns about the extend, bias, and other limitations of our pilot study. We agreed that our study could be perceived as a first approach, especially in a sanitary context, and that lacks evidence-based mHealth interventions. We believe that these findings warrant further research as the second phase of this study that includes a larger sample size and different participating centers.

*In the introduction, the authors need to introduce why FeverDX is particularly suitable to improve the adherence to CPGs and surveillance, who is it designed for and how are mhealth tools particularly suitable (something novel is not necessarily better). Some of this information is already in the discussion, but I think it would help construct the argument better if placed in the introduction (for example, lines 208-215 and lines 216-219).*

We appreciate this suggestion. We have edited the introduction section and included the following text in lines 123-131 of the file “Manuscript with track changes”:

“FeverDx is a mHealth tool designed by a group of physicians for healthcare professionals in a local context where digital transformation is limited, making this tool tailored to the specific needs of the end-user in terms of time and ease of use, access to content and particularly in a critical step of the reporting process of mandatory notifiable diseases. This particular feature could potentially improve the currently manual registration and report process, mostly using physical resources (paper forms, physical files)[10]. Moreover, the mobile app provides rapid access without an internet connection to locally stored clinical information regarding the main topics of frequent fever syndromes clinical guidelines”.

*Moreover, the whole argument is centered around that performance evaluations have not been performed for mhealth interventions (lines 69-70), which is not entirely true. There is a whole body of literature that does exactly that, and although not all mhealth tools are validated, there are performance evaluations. The reference provided for that claim, does not support it exactly.*

We thank the reviewer for pointing out that we needed reference support about performance evaluations for mHealth interventions. We have added additional sources (Lines 133 and 153) and edited Lines 111-131 in the “Manuscript with track changes” file with the following text.

Although mHealth performance evaluations are growing[12–14], regarding the majority of commercially mobile health applications in our research field, companies and researchers have not yet incorporated an extensive performance evaluation, including usability and app quality. Furthermore, additional tailored and rigorous evaluation studies on the mHealth clinical impact and reliability are still required[15,16] .

*In Methods, I would suggest making the first paragraph describing the mobile app clearer. As I continued reading, I understood the app but it is very confusing at the beginning. I would make Fig 2, Fig 1 instead. However, I am most worried about the usability evaluation. Did it happen during the training session, after the simulated emergency consultation? Or some time after? Did the authors measure actual engagement after the training session? I worry that the high scores are influenced by courtesy bias, apart from the selection bias already mentioned by the authors. Besides, the sample size is too small to even speculate about the external validity of the results.*

We agree that the mobile app description might appear confusing. We reviewed the drafting and structuring of the section, where we describe the general and functional characteristics of the mobile application. Therefore, we reorganized almost the entirely Mobile application section (Lines 171-227). We included reviewers' recommendations regarding the designation of figures 1 and 2, which we changed (Fig1 instead of Fig 2) in the "Manuscript with track changes." Moreover, the features and functionality of each application module were described separately.

Reviewer's comments regarding the usability evaluation allowed us to include the following text (Lines 237-242) to make more precise the process described in the study procedures section.

"The usability evaluation was carried out in a single session according to the following stages:

1. Delivery of materials (devices, medical records, evaluation forms) and instructions about the application use and evaluation process

2. Simulated emergency consultation (Interaction with the application)

3. Individually and confidentially usability evaluation by GPs"

Thank you for pointing out the engagement measure, which is an essential part of the evaluation and was included in the usability evaluation and refers to sections B (user engagement) and F (subjective quality assessment) in the evaluation form (included as Supporting information).

Although the usability evaluation was performed by each GP anonymously, individually and secretly, we shared the reviewer's concern about the possible introduction of a courtesy bias as a disadvantage of this approach [8]; therefore, we included it as a limitation of our study (Lines 490-493). As a strategy to reduce the risk of courtesy bias, further research could include different assignment strategies for the evaluation, such as the use of digital channels or the use of evaluation tools embedded in the mobile application as the log reporting system.

As a recognized limitation, the sample size and extent of the study should be tackled towards a further stage of clinical impact evaluation. Despite these limitations, study results can be considered as a potential strategy to support GPs in primary healthcare centers, rural areas with a limited internet connection.

*In the results:*

*How was CPG knowledge measure?*

*In lines 164-165 the authors mention that the majority of the GPs agreed that the app matched the CGP information, but in the previous sentence that almost half actually read them… so that could be also indicating courtesy bias?*

The first two questions of the evaluation form qualitatively measured CPG's knowledge (S1 Evaluation form). Nevertheless, through these questions, we gathered a general observation regarding the GP's knowledge level of Ministry of Health’s guidelines for vector-borne disease management. Since our findings revealed a surprising proportion of GPs with a lack of knowledge, we decided to include them as part of the evaluation.

Regarding the GP's perception of the app's content and GCPs agreement, we have clarified in lines 359-361 these results the proportions discriminated by subcategories with the following text:

"The proportion of GPs who reported having more comprehensive knowledge about the guides was 9/20, more than half (5/9) ultimately agreed, and 4/9 partially agreed that the app matched the CPGs."

Based on the evaluation form (S1 Evaluation form) the answer options for the first question included the multiple levels of knowledge about CPGs. The second question's option "Not applicable" was included for those who answered in the first question that they did not know the GCPs, which may have generated confusion among some evaluators. Therefore, we hope that including evaluation form facilitates results interpretation.

In the discussion, the first sentence speaks about the novelty of the study but uses a very restrictive situation so, yes it is novel for that…

We appreciate this comment. We agreed that we present this study under a narrow perspective. However, we wanted to frame the discussion around essential conditions to considerer for the development and evaluation of mobile health interventions by adding the following text in lines 440-446 of the "Manuscript with track changes" file.

"There are relevant conditions to be considered for the mobile health applications development and adoption: the local demographic, geographic, connectivity, culture, and socio-sanitary aspects; and the particular needs of the potential end-users, general public, healthcare workers or surveillance authorities[34]. Among the commercially available mHealth developments, a small proportion combines different functionalities such as vector-borne disease management (evaluation, classification, and treatment) or rapid offline clinical information review."

Also, I do agree that more usability and feasibility studies are needed for mHealth but is this study enough to state this? Is a one-time (not longitudinal) and with a very small sample. This limits the interpretation of the results and I worry about speculations as the one presented in lines 205-207.

We agree that a pilot study approach with the mentioned limitations could also limit the interpretation of results. With the previous text, we intended to narrow the scope of our results and suggested the potential (Lines 416-420) of FeverDx as a support tool susceptible to improvement regarding functionality throught iterative evaluation. We added the following text:

“Hence, the use of FeverDx in regions where arboviruses are endemic or hyperendemic potentially could help GPs and stakeholders in the management of acute febrile syndromes such as arboviruses infections. Although this is a pilot study, our findings are a starting point for the development of new implementation studies that allow the evaluation of application performance in different contexts and scenarios”.

In lines 219, the authors may have omitted other apps. Of the top of my head I can think of Kidenga, for example. What are the other 26 apps relevant to surveillance referring to?

We have added relevant information regarding mobile health developments vector-borne diseases related that we omitted in the revised manuscript. We mentioned the apps related to dengue, chikungunya, and Zika surveillance and some of their features (Lines 432-434). Besides, in lines 403-407, we included commercially available mHealth from the most popular online stores (Google Play Store and Apple Store) to complement the description with the following text (Lines 435-439):

"Among the commercially available mHealth developments, a small proportion combines different functionalities such as vector-borne disease management (evaluation, classification, and treatment) or rapid offline clinical information review."

Lines 236-237 seem to talk about the general public but this app is not intended for that.

We appreciate this observation, and we agree that this statement might appear out of context. We changed the original statement in lines 469-471, and included the following text:

"Finally, mobile technology offers unique opportunities to reach and engage healthcare professionals to enhance health literacy and public health authorities for real-time disease surveillance."

*Minor:*

*Lines 145-147: it is not very clear how the evaluation form was given in this sentence and Table 1 is presented in Results. I would suggest including the evaluation form as supp. material and improve this sentence.*

Regarding the use of the evaluation form, in the mentioned lines, we described the components of the evaluation form. As the reviewer suggested, we rewrite the description in lines 333-345 to offer a more accurate description. The evaluation form will be included as supporting information to clarify the description of the evaluation form components. In lines 237-240 (Study procedures section), we described the process in which the evaluation form is given to the evaluators.

*Lines 150-151: I assume that the authors referred to median and IQR as is stated in the following paragraph.*

In line 347 we referred to median and IQR

*Line 183: “as (un)acceptable”?*

Lines 391-393 (Manuscript with track changes file) in the results section refers to the evaluation form, B section (user engagement). Question 13 inquiry about the application contents and its suitability for the group of users it is intended. The options for the answer are totally unappropriated, mostly unappropriated, acceptable (selected by one GP), adequately oriented, correctly targeted. This is clarified with the inclusion of the evaluation form.

*6. PLOS authors have the option to publish the peer review history of their article (what does this mean?). If published, this will include your full peer review and any attached files*

*If you choose “no”, your identity will remain anonymous but your review may still be made public.*

*Do you want your identity to be public for this peer review? For information about this choice, including consent withdrawal, please see our Privacy Policy.*

*Reviewer #1: No*

*[NOTE: If reviewer comments were submitted as an attachment file, they will be attached to this email and accessible via the submission site. Please log into your account, locate the manuscript record, and check for the action link "View Attachments". If this link does not appear, there are no attachment files to be viewed.]*

*While revising your submission, please upload your figure files to the Preflight Analysis and Conversion Engine (PACE) digital diagnostic tool, https://pacev2.apexcovantage.com/. PACE helps ensure that figures meet PLOS requirements. To use PACE, you must first register as a user. Registration is free. Then, login and navigate to the UPLOAD tab, where you will find detailed instructions on how to use the tool. If you encounter any issues or have any questions when using PACE, please email us at figures@plos.org. Please note that Supporting Information files do not need this step.*

1.	Mohanty B, Chughtai A, Rabhi F. Use of Mobile Apps for epidemic surveillance and response – availability and gaps. Glob Biosecurity. 2019;1(2).

2.	Jahns R-G, Houck P. Mobile Health Market Report 2013-2017. 2013;116. Available from: http://www.research2guidance.com/shop/index.php/mhealth-app-performance-benchmarking

3.	Boyce SP, Nyangara F, Kamunyori J. A mixed-methods quasi-experimental evaluation of a mobile health application and quality of care in the integrated community case management program in Malawi. J Glob Health. 2019;9(1):010811.

4.	Haque F, Ball RL, Khatun S, Ahmed M, Kache S, Chisti MJ, et al. Evaluation of a Smartphone Decision-Support Tool for Diarrheal Disease Management in a Resource-Limited Setting. PLoS Negl Trop Dis. 2017;11(1):1–19.

5.	Hamrioui S, Torre-díez I De, López-coronado M, Herreros J. Implementation and Performance Evaluation of a Mobile Health Application for Auto-controlling of Cardiac Diseases. 1(1):13–7.

6.	Fougerouse P-A, Yasini M, Marchand G, Aalami OO. A Cross-Sectional Study of Prominent US Mobile Health Applications: Evaluating the Current Landscape. AMIA . Annu Symp proceedings AMIA Symp. 2018 Apr;2017:715–23.

7.	Sim I. Mobile devices and health. N Engl J Med. 2019;381(10):956–68.

8.	Hameed W, Ishaque M, Gul X, Siddiqui J-R, Hussain S, Hussain W, et al. Does courtesy bias affect how clients report on objective and subjective measures of family planning service quality? A comparison between facility- and home-based interviews. Open Access J Contracept. 2018;Volume 9:33–43.

9.	López JM, Urretavizcaya M, Losada B, Fernández I. Field vs. Laboratory usability evaluations: A study on a context dependent mobile application developed with an agile methodology. IEEE Lat Am Trans. 2016;14(1):339–48.
