# Supplementary material for: Acceptability and usability of a mobile application for management and surveillance of vector-borne diseases in Colombia: An implementation study
Source: PLoS One. 2020 May 29;15(5):e0233269. doi: 10.1371/journal.pone.0233269 (PMC7259752; doi:10.1371/journal.pone.0233269)
Supplement: Attachment — Submitted filename: Response to Reviewers_07_04_2020.docx [file pone.0233269.s005.docx]

Editors

*PLOS ONE*

April 28^th^, 2020

Dear Dr. Samy,

We thank you and the reviewers for a thorough revision and valuable suggestions regarding our manuscript and for the opportunity to revise and resubmit. We are pleased to submit the improved manuscript. On the following pages, we include our response to each recommendation and comments. On behalf of my co-authors, I thank you for your consideration of this resubmission. We appreciate your time and look forward to your response.

Sincerely,

Sarita Rodríguez R, M.D. (corresponding author) sarita.rodriguez@fvl.org.co, +573173820407

*PONE-D-19-31941R1
Acceptability and usability of a mobile application for management and surveillance of vector-borne diseases in Colombia: An implementation study
PLOS ONE

Dear Dr. Rodriguez,

Thank you very much for submitting your manuscript "Acceptability and usability of a mobile application for management and surveillance of vector-borne diseases in Colombia: An implementation study" (#PONE-D-19-31941R1) for review by PLOS ONE. As with all papers submitted to the journal, your manuscript was fully evaluated by academic editor (myself) and by independent peer reviewers. The reviewers appreciated the attention to an important health topic, but they raised substantial concerns about the paper that must be addressed before this manuscript can be accurately assessed for meeting the PLOS ONE criteria. Therefore, if you feel these issues can be adequately addressed, we invite you to submit a revised version of the manuscript that addresses the points raised during the review process. We can’t, of course, promise publication at that time.*

*We would appreciate receiving your revised manuscript by May 02 2020 11:59PM. When you are ready to submit your revision, log on to https://www.editorialmanager.com/pone/ and select the 'Submissions Needing Revision' folder to locate your manuscript file.

If you would like to make changes to your financial disclosure, please include your updated statement in your cover letter.

To enhance the reproducibility of your results, we recommend that if applicable you deposit your laboratory protocols in protocols.io, where a protocol can be assigned its own identifier (DOI) such that it can be cited independently in the future. For instructions see: http://journals.plos.org/plosone/s/submission-guidelines#loc-laboratory-protocols

Please include the following items when submitting your revised manuscript:*

- *A rebuttal letter that responds to each point raised by the academic editor and reviewer(s). This letter should be uploaded as separate file and labeled 'Response to Reviewers'.*
- *A marked-up copy of your manuscript that highlights changes made to the original version. This file should be uploaded as separate file and labeled 'Revised Manuscript with Track Changes'.*
- *An unmarked version of your revised paper without tracked changes. This file should be uploaded as separate file and labeled 'Manuscript'.*

*Please note while forming your response, if your article is accepted, you may have the opportunity to make the peer review history publicly available. The record will include editor decision letters (with reviews) and your responses to reviewer comments. If eligible, we will contact you to opt in or out.*

*We look forward to receiving your revised manuscript.

Kind regards,

Abdallah M. Samy, PhD
Academic Editor
PLOS ONE*


*Reviewers' comments:

Reviewer's Responses to Questions

Comments to the Author

1. If the authors have adequately addressed your comments raised in a previous round of review and you feel that this manuscript is now acceptable for publication, you may indicate that here to bypass the “Comments to the Author” section, enter your conflict of interest statement in the “Confidential to Editor” section, and submit your "Accept" recommendation.

Reviewer #1: All comments have been addressed
Reviewer #2: (No Response)*

*2. Is the manuscript technically sound, and do the data support the conclusions?

The manuscript must describe a technically sound piece of scientific research with data that supports the conclusions. Experiments must have been conducted rigorously, with appropriate controls, replication, and sample sizes. The conclusions must be drawn appropriately based on the data presented.

Reviewer #1: Partly
Reviewer #2: Partly*

*3. Has the statistical analysis been performed appropriately and rigorously?

Reviewer #1: Yes
Reviewer #2: N/A*

*4. Have the authors made all data underlying the findings in their manuscript fully available?

The PLOS Data policy requires authors to make all data underlying the findings described in their manuscript fully available without restriction, with rare exception (please refer to the Data Availability Statement in the manuscript PDF file). The data should be provided as part of the manuscript or its supporting information or deposited to a public repository. For example, in addition to summary statistics, the data points behind means, medians and variance measures should be available. If there are restrictions on publicly sharing data—e.g. participant privacy or use of data from a third party—those must be specified.

Reviewer #1: Yes
Reviewer #2: No*

We appreciate this observation; thus, to compliant the PLOS Data Policy, we will make raw data concerning the results of the FeverDx usability and acceptability evaluation that supports our work fully available as part of the supporting information (S2 Dataset)

*5. Is the manuscript presented in an intelligible fashion and written in standard English?

PLOS ONE does not copyedit accepted manuscripts, so the language in submitted articles must be clear, correct, and unambiguous. Any typographical or grammatical errors should be corrected at revision, so please note any specific errors here.

Reviewer #1: Yes
Reviewer #2: Yes*

*6. Review Comments to the Author

Please use the space provided to explain your answers to the questions above. You may also include additional comments for the author, including concerns about dual publication, research ethics, or publication ethics. (Please upload your review as an attachment if it exceeds 20,000 characters)*

***Reviewer #1:*** *The authors have responded to my concerns in a satisfactory way and although my concerns about bias and external validity remains, they have been able to make those limitations more explicit. They have greatly improved the manuscript, although minor changes need to be addressed before I can recommend it for publication.*

***Abstract*** *1- In the background it says: “A large number of mHealth tools are available; however, very few have been evaluated regarding usability and acceptability.”
This is not appropriate as discussed in the previous review and addressed in the reviewer’s comments. Look at the second paragraph of your introduction and use that argument. Why mHealth can help address the problem*.

Thank you for this suggestion. We modified text in the file “Manuscript with track changes”.

*2- In the results is says “Seventy-five percent of the evaluators reported being aware of the Colombian Ministry of Health’s guidelines.”
This is not in the results though, either include in the results or only indicate that less than half had comprehensive knowledge (see comments on the CPG knowledge section of results).*

Thank you for pointing this out. We followed your suggestion and modified accordingly in the results section.

*3- In the conclusion it says “Despite a large number of mHealth tools available, the literature lacks evaluated and evidence-based mobile technology.” This is not completely true as described previously. The new discussion section address this pretty well, so you need to revisit that argument here and match the discussion.*

*4- Conclusions section is too general, you need to answer why FeverDx could help advance or improve practice based on the results of the pilot study. Also briefly mention the limitations (e.g., although it was a pilot study with a small sample size, FeverDx…)*

Thank you for these observations, we substantially modified the conclusions section in the abstract and some of the study limitations were also mentioned in the conclusion section as follows:

“FeverDX, a mobile application, is a novel mHealth strategy to strengthen care processes and facilitate the detection and reporting of notifiable surveillance diseases. It could improve adherence to clinical practice guidelines for the management and prevention of prevalent diseases as arboviruses in healthcare settings.

Although this pilot study used a small sample size, FeverDx performed adequately in a simulated emergency consultation. Further implementation studies are needed to increase the reliability of mHealth technologies in different scenarios.”

***Introduction*** *5- Where it says “…time and ease of use, access to content and particularly in a critical step of the reporting process of mandatory notifiable diseases.” Change for “…time and ease of use, and access to content in a critical step of the reporting process of mandatory notifiable diseases.”*

Thank you for this suggestion. We agreed this modification notably improve the understanding of the text.

*6- Where it says “This particular feature, we hypothesize that could potentially improve the currently manual registration and report process, mostly using physical resources (paper forms, physical files) [10].” Which feature are you referring to? Maybe rephrase it as: “We hypothesize that these features (ease of use and rapid access to content) can potentially…”*

We thank you for this observation, and we agreed that the sentence after the period is unclear. We are referring to the feature regarding the reporting process.

*7- Where it says “There are a recently increasing supply and demand of mobile applications development in the healthcare area”. Change for “Supply and demand of mobile applications development in the healthcare area have been recently increasing.”*

We appreciate this suggestion. We adopted this change in the “Manuscript with track changes”.

*8- Where it says “This study evaluates the usability and acceptability of FeverDx…” Briefly mention who valuated it and where. Use the first sentence from Methods.*

We appreciate this suggestion, we included additional information regarding the evaluators and the study setting in the “Manuscript with track changes”.


***Methods***

*9- Where it says “Moreover, an infectious disease specialist reviewed the included recommendations.” Moreover can and should be removed.*

We removed “Moreover” of the “Manuscript with track changes” file.


*10- Where it says “Obtained data from registration, evaluation and reporting is storage” Change storage for stored.*

We changed “storage” for “stored” in the “Manuscript with track changes”.


*11- The following phrase is confusing: “The evaluation form comprises a 25 item measure that includes four objective quality - information quality, engagement, functionality, aesthetics, and two subjective subscales-quality and impact.”
All items are subjective in the strict sense, based on the S1 File provided, I would recommend that you rephrase it as:*

*“The evaluation form comprises 25 questions measuring quantity and quality of information (Section A), engagement (Section B), functionality (Section C), aesthetics (Section D), impact of patient management from the practitioner perspective (Section E) and acceptability by health practitioners (Section F) (S1 file).”*

Thank you for this helpful recommendation; we rephrase the entire paragraph.


*12- Where it says “The evaluation test is scored by determining the mean scores of the app quality subscales and the total mean score.” You should rephrase it as: “A global score was obtained for the evaluation form test by determining the median scores of each subsection.”*

Thank you for this observation. We edited the text in the “Manuscript with track changes”, rectifying the used measure (median).

*13- S1 file: I suggest some changes:

Seccion E: the brief description is the same as Seccion D. This section measures impact on patient management from the practitioner perspective.*

*Seccion F: This section measures acceptability of users. Include this in the description.*

We appreciate this suggestion. We edited the evaluation form (S1 file), including the modified description in section E, and added a description for section F.

***Results***

*14- In the CPG knowledge section, to improve readability, I suggest rephrasing the whole section as:
“The proportion of GPs with comprehensive knowledge of the Colombian’s CPG for diagnosis and management of arboviruses (dengue, chikungunya, and Zika) was less than half of GPs evaluated (9/20) and less than one third of them applied them in their practice (6/20). Of those GPs who reported having comprehensive knowledge of CPG, more than half (5/9) wholly agreed and, 4/9 partially agreed that the application information matched with CPG information.”*

We thank you for this valuable suggestion; we edited the entire paragraph in the results section (lines 420-426) in the “Manuscript with track changes” as follows:

All data regarding CPG knowledge evaluation was gathered from GPs with no prior use of the FeverDx app. The proportion of GPs with comprehensive knowledge of the Colombian’s CPG for diagnosis and management of arboviruses (dengue, chikungunya, and Zika) was less than half of GPs evaluated (9/20), and less than one-third of them applied them in their practice (6/20). Of those GPs who reported having comprehensive knowledge of CPG, more than half (5/9) wholly agreed and, 4/9 partially agreed that the application information matched with CPG information.

*15- Table 1: change subjective assessment of quality by acceptability*

Thank you for this observation. We edited Table 1, the last category name by “acceptability.”

*16- Where it says “Regarding the "interest generated by the application" item, GPs scored a median of 5 (IQR=4-4.5).”, which section is this in the table? Be consistent with names. Also the median doesn’t match the IQR, check that.*

We thank the reviewer for pointing out that we needed to be consistent with the category names. We edited the “Manuscript with track changes” (lines 451, 452) where we were referring to the “user feedback” category and we corrected the median score. The text is included as follows:

“Regarding the user feedback, GPs scored a median of 4 (IQR=4-4.5), and “interest” obtained the high scored of this category (5 (IQR =4-5)”

*17- Where it says “Moreover, all the GPs recommended the app”, change it for “Moreover, all the GPs would recommend the app”*

Thank you for this recommendation, we edited the “Manuscript with track changes” in line 464 with the following text:

“Moreover, all the GPs would recommend the app, and most of them (17/20) rated it with 4 out of 5 according to the overall star rating (Fig 4).”

***Discussion***

*18- Where it says “Mobile applications designed for epidemiological surveillance of arboviruses are scope limited”, rephrase it as: “Mobile applications designed for epidemiological surveillance of arboviruses are limited in their scope”*

Thank you for this observation. We modified the “Manuscript with track changes” (lines 498 and 499) accordingly.

*19- Where it says ”On the one hand, a study conducted in 2018”, “On the one hand” is not appropriate. You should remove it.*

Thank you for this recommendation. We deleted “On the one hand” from the “Manuscript with track changes”.

*20- Where it says “...that aimed case reporting and outbreaks..”, change for aimed at case…*

Thank you for this suggestion. We edited the “Manuscript with track changes”.

*21- Where it says “…some limited by country, the language with narrow applicability…” Do you mean that the language limited their applicability because they were mostly in English? Were they designed to be used by English-speaking users or in developed countries? Making that more explicit will help your argument.*

We appreciated this observation. We changed the original statement, and included the following text:

Some of these applications were limited by location, operating system (Android only), and although most of them were developed for global use, most of them were in the English language, which could narrow their applicability in different contexts

*22- Where it says “However its implementation in clinical scenarios is scarce”, do you mean they are not designed to be used by GPs in clinical scenarios? Rephrase accordingly.*

Thank you for this recommendation. We edited the original sentence to clarify that, although some of the mentioned mHealth are intended for healthcare professionals use, evidence regarding the actual implementation in clinical settings is scarce. We modified text in the “Manuscript with track changes” as follows:

“Additional mHealth developments with a different approach are available such as surveillance (DengueWatch, Dengue map, ZikaTracker, Kidenga App, Break Zika, Predict and Beat dengue) and general information (Dengue – Manejo clínico, Arbo App, Dengue App, Dengue síntomas tratamiento y prevención, Dengue Treatment, WHO Zika App), however, evidence regarding implementation in clinical scenarios is scarce.”

*23- Where it says “A striking find was”, change for “A striking finding was”*

We appreciate this observation. We edited the “Manuscript with track changes”.

*24- This phrase “…although the majority of the GPs agreed that the application content was according to management guidelines.” is not necessary here and interrupts the flow. Moreover, using although implies that is in contrast with the previous statement which is not. I would remove this phrase altogether.*

Thank you for this suggestion. We removed the statement entirely

***Limitations***

*25- Consider including a statement about their applicability mostly restricted to Colombia, given the country specific CPG and surveillance systems.*

Thank you for this suggestion. We included a text regarding the restricted applicability in the “Manuscript with track changes” as follows:

“Third, the implementation of FeverDx is currently restricted to Colombia because its design was mainly based on the Colombian Ministry of Health´s mandatory notification sheets and CPG. Application content was strengthened with the international recommendations given by the WHO and with the review by an infectious diseases specialist with experience in arboviral research to mitigate this issue.”

***Reviewer #2:*** *Even though the article is clear and the mobile app is interesting the findings does not correspond to an orignial article, it is more suitable for a short communication.*

We appreciate the positive comments and the efforts by you and the reviewers on the manuscript. Although co-authors and myself are aware of further research is needed for the mobile app clinical validation, we respectfully consider that our work could be a foundation to the mHealth implementation in the infectious diseases field and the clinical context in tropical countries such as Colombia. We believe it is essential to present the main characteristics of the application and more in-depth the process of implementing this mobile application evaluation methodology since the evidence related to the evaluation of the usability and acceptability of mobile applications is still a challenge for developers, researchers, and healthcare professionals involved.

***Abstract:*** *in methods I would add the grade scale of uMARS, if not results are not understandable without reading the full article.*

We appreciate this valuable recommendation. We edited the methods section.

*“A pilot implementation study was conducted to evaluate the usability and acceptability of FeverDX based on the modified version of the Mobile Application Rating Scale (uMARS). The evaluation form included 25 questions regarding quantity and quality of information, engagement, functionality, aesthetics, impact, and and acceptability by healthcare workers. Each item uses a 5-point scale (1-Inadequate, 2-Poor, 3-Acceptable, 4-Good, 5-Excellent). A global score was obtained for the evaluation form test by determining the median scores of each subsection. A descriptive statistical analysis of the data obtained was performed.”*

***Introduction:*** *it is not clear which is the potential impact of the using of the app among GP. Line 67, this paragraph should be at the end of the introduction or even in methods.*

Thank you for this observation, we moved the paragraph at the end of the introduction.

***Methods:***
*Fig2, when referring to alarm signs that includes all dengue warning signs? if so it should be stated.*

Thank you for this observation. In Fig 2, we summarize the flow of care using FeverDx. This algorithm highlights the essential points of patient care, such as the evaluation of general symptoms, vital signs, and alarm signs, severity signs, among others. Within the app in the view "alarm signs," all dengue, Zika, and chikungunya warning signs were included. We included the following statement:

“This algorithm highlights the essential points of patient care, such as the evaluation of general symptoms, vital signs, and the alarm signs, severity signs, which includes all dengue, Zika, and chikungunya warning signs.”

***Results:*** *CPR knowledge prior of after using the app?
It is not clear if the app was used in clinical context, with how many patients, if it was useful for the final diagnosis.*

We appreciate this observation. CPG knowledge evaluation was measured by the first two questions of the evaluation form (S1 Evaluation form). Through these questions, we gathered a general observation of the GP's knowledge level, prior of using the app (study procedures section in line 425), regarding Ministry of Health’s guidelines for vector-borne disease management. Nonetheless we agreed this statement could be included in the CPG Knowledge sub-section with the following text of the "Manuscript with track changes" file:

*“All data regarding CPG knowledge evaluation was gathered from GPs with no prior use of the FeverDx app. The proportion of GPs with comprehensive knowledge of the Colombian’s CPG for diagnosis and management of arboviruses (dengue, chikungunya, and Zika) was less than half of GPs evaluated (9/20), and less than one-third of them applied them in their practice (6/20). Of those GPs who reported having comprehensive knowledge of CPG, more than half (5/9) wholly agreed and, 4/9 partially agreed that the application information matched with CPG information.”*

The FeverDx app was used during a simulated ER consultation with no patients. The GPs used clinical information from 100 patients (medical records, laboratory reports, among others) who had previously consulted the hospital, all (100) cases were reported (real-time report feature). After the GPs-App interaction, GPs performed FeverDx evaluation, as described in the methods section. We are aware that further research is needed to validate the clinical impact of using the mobile app, which includes the usefulness in the final diagnosis.

***Discussion:*** *there is no future plans for further research or for the app.*

Thank you for this observation. We share your concerns about the extent of our pilot study. We believe that our study could be perceived as a first approach, especially in a sanitary context, and that these findings warrant further research as the second phase of this study that includes the clinical impact evaluation with a larger sample size and different participating centers. We included the following text at the end of the limitations section in the “Manuscript with track changes”:

*“Finally, further research with a larger sample size and with the participation of other healthcare centers need to be conducted to support our initial findings.”*

***General thoughts:*** *First of all, it could be missleading using an app for fever which do not discriminate malaria or bacterial infections at the begining. An algorithm of fever in the tropics should rule out the most dangerous causes first, and if it is not a malaria zone it should be stated; nevertheless bacterial infections are distributed worlwide.*

*Although the info about this app should be shared with the community little information could be extracted from this article. I would suggest to compare two GP groups, one using the app and the other not using it and seeing the differences; but the most important thing is that the app is not validated with confirmed cases, apparently the purpose of the app is only to apply the guidelines, if it is so it should be explained. Could be much more interesting if this app can be used by health care workers who not are doctors. Maybe the app should focuse more in epidemiological vigilance.*

We are grateful for your appreciation, and, respectfully, we would like to answer your concerns about our work. FeverDX is an app developed with three main objectives:

- Evaluation and patient management support when there are cases suspected of vector-borne diseases.
- Inform the user about general aspects regarding frequent infections found in tropical countries such as Colombia.
- Support local surveillance systems allowing real-time reporting of suspected cases.

FeverDX’s design does not intend to make a specific diagnosis as a primary objective, but to provide support to GPs to do a differential diagnosis considering the time of onset of symptoms and clinical characteristics through the information based on the CPGs that are contained in the clinical guideline module of the app. In this sense, FeverDx it is not designed as a mobile diagnostic app; however, it includes information regarding the possible differential diagnosis such as malaria, leptospirosis among others prevalent febrile syndromes in tropical areas such as Colombia. Either in the clinical guideline module or the feedback messages in the evaluation module FeverDx includes clinical information to support the physician’s diagnostic and management process. For this reason, the opening app disclaimer recommends prioritizing the clinician criteria over the app recommendations.

Additionally, after the patient’s clinical data laboratory results are registered in the app, it provides feedback to the GPs regarding the patient’s management according to the risk group and clinical evaluation. Finally, FeverDX allows GPs to do real-time reporting of the suspected or confirmed case, including georeferenced information, that is stored in a secured server at the hospital’s data center, providing safe access to the epidemiological surveillance authorities.

Although in this pilot study, the application evaluation was only conducted with GPs, FeverDX was designed to be used by healthcare workers (medical and non-medical). Considering that Colombia is hyperendemic for arbovirosis and the overload of the healthcare system generated by these diseases, its innovative use in rural and disperse communities with limited health care service access could enable healthcare workers located in this areas, where medical availability is reduced, to perform the evaluation and patient management. In this sense, FeverDX, a user-centered tool, could eventually avoid under-registration of cases by using the real-time case report feature.

We shared your concerns regarding FeverDx clinical validation.

We proposed this study as an initial phase of performance evaluation. However, we are aware of our limitations this retrospective evaluation was performed with 100 clinical charts from confirmed cases (dengue, zika and chikungunya positive) with the constant support of an infectious disease specialist and medical advisory team.

The comparison between two different groups is an attractive study design to be implemented for clinical validation. However, we respectfully consider that this should be evaluated in further research since the main objectives of our work and this manuscript were (1) to describe and introduce the main FeverDX’s functionalities, (2) to present the usability and acceptability evaluation by healthcare workers (medical doctors at a clinical setting.
